# New Appliances and Things Medical

**Published:** 1907-11-23

**Authors:** 


### New Appliances and Things Medical.

[We shall be glad to receive at our Office, 28 & 29 Southampton Street, Strand, London, W.C., from the manufacturers, specimens of all new preparations and appliances which may be brought out from time to time.]

### NEW PREPARATIONS.

(Evans, Gadd and Co., Ltd., 23 and 24 Redcliff Street, Bristol.)

We have received from this well-known firm of manufacturing chemists samples of three new preparations. Of these Unguentum retlanol (Gadd) is a sedative and antipruritic ointment which has been found beneficial in cases of irritation, and in one case of so-called lysol rash was found to be decidedly soothing. Unguentum ranunculi ficariæ (Gadd) is an ointment which has as its chief ingredient one of the active principles of the lesser celandine (pilewort)—a plant which has been reputed of use in the treatment of hæmorrhoids, and which has lately been reintroduced into favour. There is already a pilewort ointment in the Pharmacopœia, made by digesting the dried plant with melted lard, but this new preparation, which is apparently compounded with lanoline, seems to be superior to the Pharmacopœial mixture. The third novelty is the Liquor thymo-antiseptic co. (Gadd), which is a powerful, non-irritating, deodorant and antiseptic. All three preparations merit further trial at the hands of the practitioner.

### COAL-TAR PREPARATIONS.

(Wright, Layman and Umney, Ltd., Southwark, London, S.E.)

Wright's coal-tar specialities have received such spontaneous and such general appreciation from the profession that it would be a work of supererogation to enumerate their good qualities. Those who have not tried them should write for samples directly to the firm, and give the various preparations a thorough test, the result of which, we feel confident, will be eminently satisfactory. The liquor carbonis detergens, which is in reality an alcoholic solution of coal-tar, is an elegant preparation of striking efficacy in the treatment of irritative skin lesions, and its only rivals are the liquor picis carbonis and the much less well-known tinctura licanthracis. The sapo carbonis detergens, popularly known as "Wright's Coal-tar Soap," is familiar enough to every housewife, and is deservedly one of the most popular toilet soaps with medical men. It is a superfatted soap possessing strong antiseptic properties. The samples which we have lately received from the firm show no deterioration in the manufacture or preparation of these specialities, which continue to maintain their high standard of excellence.

### THOMPSON'S APPLE TEA.

(Pure Food Speciality Co., 21 Fieldhouse Road, Balham, S.W., late 8 Hart Street, Bloomsbury.)

This is a powder stated to be made from choice and selected apples. It is intended to be used as a substitute for tea or coffee by persons suffering from gout, rheumatism, sciatica, diseases of the liver and kidneys, headache, etc. Care is taken in the preparation of the apples by this firm that the valuable acids contained in the fruit are preserved, while in order to take advantage of the nutritive qualities of the peel, an extra amount of the latter is used in the manufacture of the powder. The tea is easily made, and by the addition of a little milk, cream, or lemon, a palatable drink is the result, which should prove a useful beverage for those with a tendency to uric acid deposits.

